# The Importance of Intergenerational Leadership Praxes and Availability of Key Information for Older Employee Burnout and Engagement in the Context of Firm Size

**DOI:** 10.3389/fpsyg.2022.858134

**Published:** 2022-04-12

**Authors:** Maja Rožman, Borut Milfelner

**Affiliations:** Faculty of Economics and Business, University of Maribor, Maribor, Slovenia

**Keywords:** leadership, burnout, work engagement, older employees, firm size

## Abstract

The main aim of this study was to analyze the effects of availability of key information and intergenerational leadership on burnout divided into physical symptoms of burnout and emotional symptoms of burnout and work engagement regarding the firm size during the coronavirus disease 2019 (COVID-19). The empirical study included 583 older employees in Slovenia who participated in the survey during the COVID-19 pandemic. Structural equation modeling was used to explore the effects between constructs. We analyzed structural paths for the entire sample and for the two groups (small and large companies). According to the results concerning both groups, the impact of the availability of key information on emotional burnout is negative only for small companies. Contrary to that the negative impact of intergenerational leadership on emotional burnout is much stronger in large companies. Concerning the impact of physical burnout on emotional burnout, the positive impact of physical burnout exists in both types of companies but is stronger in small companies. The findings will contribute to a clearer picture and the adoption of further measures to prevent burnout in the workplace and increase work engagement concerning the firm size, especially during the COVID-19 pandemic.

## Introduction

Creating an appropriate work environment contributes to employees’ wellbeing, health, and work motivation (Bakker et al., 2014). Therefore, companies need to consider demographic change and create jobs to adapt to an aging workforce (Parker, 2014). Companies need to be aware that older employees in good health and wellbeing are more engaged and more motivated to continue working (Ekoh, 2021). Some authors identify three age groups of older employees: the first group is younger aging employees, aged from 45 to 54; the second group is medium aging employees, aged from 55 to 65; and the third group includes older aging employees, aged more than 65 (Tew, 2004). In most cases, the lower age limit defining older employees is 45 years (Brooke, 2003) or 50 years (Ilmarinen, 2001). Ghosheh et al. (2006) argued that the term “older employees” includes workers between 40 and 50 years of age. Older employees who work in organizations that support demographic changes, human resource practices tailored to their needs and values, and support a positive development climate are more engaged in the workplace (Adisa et al., 2021). Older employees who do not have opportunities for growth and development in the workplace, autonomy, and social support are less engaged to continue working (Lichtenthaler and Fischbach, 2016a). Hence, appropriate job design contributes to employees’ wellbeing, health, and work engagement (Bakker et al., 2014). Therefore, companies need to constantly design jobs to adapt jobs to an aging workforce (Adisa et al., 2021), which is particularly important during the coronavirus disease 2019 (COVID)-19 pandemic (Ekoh, 2021).

According to Eurobarometer data, in 2019, 92% of respondents in Slovenia were satisfied with their lives, which is more than the EU average (84%). At the time of COVID-19 and restrictive measures, Slovenia’s average assessment of life satisfaction was slightly lower than the EU average (Institute of Macroeconomic Analysis and Development, 2020a). The COVID-19 pandemic has brought indescribable changes to our daily routine. Many companies and organizations were temporarily or permanently closed during this time, and employees are shifting to remote and virtual work environments (Ratten, 2021). Many occupations that work from home were not previously envisaged had to adapt to change. Such a situation has a significant and negligible impact on employees’ wellbeing, thinking, and health (Pataki-Bittó and Kapusy, 2021).

The increase in mental health problems is typical of all developed countries due to fast-paced lifestyles, high expectations of the individual, unhealthy lifestyles, growing inequalities, disadvantages, and loneliness of the elderly. During the COVID-19 epidemic, the situation worsened. The COVID-19 epidemic directly impacts people’s stress and anxiety (Institute of Macroeconomic Analysis and Development, 2020b). Changed lifestyles (social isolation and loneliness, distance learning, work at home), reduced opportunities for a healthy lifestyle, concerns about employment and income, and additional overburdening of the health system can affect people’s mental and physical health during the COVID-19 epidemic (Kaptangil, 2021). Restrictive measures to curb COVID-19 have led to unexpected job closures worldwide and thus to changed forms of work, and the cessation of economic activity has threatened many jobs (Lamprinou et al., 2021). The pandemic has accelerated the process and forced many companies to be more agile and decentralized and agile leadership is becoming more important (McCombs and Williams, 2021). The benefit of such praxes is shown in the ability to be calm in the face of pressure, open to innovation, and able to keep teams grounded and on the right track (Attar and Abdul-Kareem, 2020).

During this time, business arrangements are changing in light of the global pandemic of COVID-19. Employee engagement is an attitude that makes all employees commit to their organization’s goals and values (Chanana and Sangeeta, 2020). Vogelgesang et al. (2013) and Guérin-Marion et al. (2018) found that appropriate leadership positively impacts work engagement among employees and proper administration reduces burnout among employees. According to Sarangi and Nayak (2016), the availability of key information, trust, and good communication between the company and employees are essential. This unification between the employee and the company is a necessity as both can achieve the best business results. Chandani et al. (2016) reported that work engagement is based on reliability, belief, commitment, communication, and key information between a company and its adherents. The companies can increase work engagement by improving senior leadership’s decision-making, responsibility, and transparency. Furthermore, Garg et al. (2017) found that a higher level of employee engagement leads to less absenteeism, emotional burnout symptoms, physical burnout symptoms, better health, and wellbeing. Moreover, a study shows that employees’ work engagement has an effect on a company’s bottom line and is strongly related to business performance (Saks, 2017). However, there is still rare evidence of how older employees have adapted to the challenges of new working practices during the COVID-19 epidemic and how the agile leadership practices could potentially lower the risk of burnout and increase the work engagement of older employees. In addition, the concepts of leadership (key information), burnout, and engagement have rarely been explored in the elderly population during the COVID-19.

During the epidemic, high-quality reconciliation of work and family responsibilities is a major challenge for employees. Workers who do their paid work as usual during this time are more concerned about their health exposed to stress. However, when performing work at home, which in normal circumstances could help to facilitate the reconciliation of professional and family obligations, workers in these situations may be burdened with challenges such as the absence of network connections or the performance of tasks in a concise time (McCombs and Williams, 2021). Regardless of the way of working, workers in private life face additional burdens due to custody obligations and distance schooling of children. The results of the all-Slovenia COVID-19 research insight were mainly focused on specific demographic groups and show that 40% of parents rated distance learning as stressful. They spent an average of 140 min a day helping their youngest primary school pupil complete school obligations, and 74% estimated that they spent more time helping pupils complete school obligations than before the outbreak of COVID-19. Single parents can be even more challenged, especially when they do not have the possibility of informal childcare (European Institute for Gender Equality, 2020).

Several studies have examined leadership (Cong and Thu, 2021), employees’ wellbeing (Gregersen et al., 2016), motivation, creativity, engagement (Bojadziev et al., 2019; Knezović and Drkić, 2021), and stress (Bean, 2018) from the viewpoint of the firm size. But according to our knowledge, the studies have not yet explored how the specific agile leadership practices from the perspective of providing the key information to employees can influence the presence of burnout and work engagement specifically for older employees during the COVID-19 pandemic, also considering the mediation role of the company size. This variable could potentially be important since the employees of smaller companies seldom benefit from strong wellbeing programs targeted at specific groups of employees simply because of a lack of capital or because of their organizational systems (Baron and Markman, 2003).

Structural equation modeling using group comparisons has been proven to help examine the effects between constructs; therefore, this methodology is used in our study. The multidimensional model includes burnout divided into physical and emotional symptoms, availability of key information, intergenerational leadership, and work engagement, whereas the firm size was used as the mediating variable.

## Literature Review and Hypotheses

### Burnout and Symptoms of Burnout of Employees During the COVID-19 Pandemic

Work is one of the most critical areas in our lives as it provides an individual with existential security, perfects him, provides a social network, and provides self-esteem and self-confidence, but it can be a source of dissatisfaction and negative influences (such as work burnout) (Mustafa, 2015). Burnout causes exhaustion and overwork in the workplace and consists of three components: a tendency to depersonalize others, emotional exhaustion, and weakened perceptions of achievement in the workplace (Maslach et al., 2001). According to Ruotsalainen et al. (2015), emotional exhaustion means feeling a lack of energy and awareness due to unbalanced demands. World Health Organisation (2019) defined job burnout as a syndrome resultant from prolonged workplace stress, with symptoms including exhaustion, reduced work efficiency, increased mental distance from work, and negative feelings or cynicism associated with the workplace (World Health Organisation, 2019). Burnout may cause physical, emotional, and behavioral illnesses for employees. Physical symptoms of burnout are associated with headaches, chronic fatigue, sleeping problems, increased blood pressure, stomach pain, tiredness, exhaustion, increased risk of cardiovascular diseases, and musculoskeletal pains (Rod and Ashill, 2009). Emotional symptoms of burnout are associated with depression, anxiety, sadness, suicidal ideation, and hypersensitivity, whereas behavioral symptoms of burnout are related to lack of concentration, avoidance of activities, insomnia, reduced working capacity, lack of willingness to work, and lack of socializing with coworkers (Mosadeghrad, 2014). On the organizational level, different symptoms of burnout are linked to employee engagement and satisfaction (Schneider et al., 2017), especially during the COVID-19 pandemic (Adisa et al., 2021). Before the COVID-19, in October 2019, the World Economic Forum found out that the sheer pace and depth of transformational change in the workplace was the greatest threat to workforce health and wellbeing and driving rising levels of anxiety and declining levels of engagement (World Economic Forum, 2019). Prescient companies were already concerned about the impacts on an employee’s mental health and the potential impact on productivity and work satisfaction. The COVID-19 pandemic acted as an accelerant, making a bad situation worse (Felstead and Reuschke, 2021). Shatte’ (2021) found that between December 2019 and June 2020, the risk for burnout had increased by 9% whereas motivation and engagement were decreased by 29%. Heading off these downturns in productivity and wellbeing, a priority prior to the global pandemic, became critical. Physical symptoms of burnout have a positive impact on emotional symptoms of burnout among older employees (Pluta and Rudawska, 2021), which have a negative effect on an employee’s wellbeing, quality of worklife, health, productivity, and performance (Lichtenthaler and Fischbach, 2016a; Haar, 2021). Physical burnout symptoms, manifested in lack of energy or chronic fatigue, have a positive effect on emotional burnout symptoms, which could be manifested in depression. This has a negative impact on the work engagement of older employees (Frins et al., 2016). According to Henkens and Leenders (2010), older employees report higher burnout symptoms. They report that an increased workload, heavy physical work, lack of challenge, autonomy, and social support from colleagues and managers are related to burnout complaints. On the contrary, in favorable psychological conditions and suitable job characteristics, work engagement, and efficiency of older employees grow (Lichtenthaler and Fischbach, 2016a). Therefore, the following hypothesis is proposed:

H1: *Older employees’ physical burnout symptoms positively impact their emotional burnout symptoms.*

### Burnout and Work Engagement of Older Employees

Burnout in the workplace is a factor that should be improved to prevent the loss of quality of work, productivity, morale, and older employees’ mental or physical health (Ekoh, 2021). According to Eriksson and Lindström (2006), a sense of coherence is promoting older employee health; therefore, Vogt et al. (2013) stated that there is a negative relationship between burnout and a sense of coherence. Burnout syndrome is currently the most important work-related stress, which causes significant social and economic losses (Ekoh, 2021). Burnout in the workplace negatively affects the health of older employees and their work engagement as it can lead to many diseases such as heart disease, diabetes, headaches, and migraines (Lichtenthaler and Fischbach, 2016b). Older employees burned in the workplace are physically and emotionally exhausted and are less engaged to continue their work (Ekoh, 2021). Emotional exhaustion and a lack of work resources negatively affect employees’ energy level and health, which lead to a lower level of work engagement (Schaufeli and Bakker, 2004; Frins et al., 2016). According to this, the following two hypotheses are proposed:

H2: *Older employees’ physical burnout symptoms negatively impact their work engagement.*

H3: *Older employees’ emotional burnout symptoms negatively impact their work engagement.*

### Availability of Key Information and Intergenerational Leadership

The field of information technology is evolving with incredible speed (Santana, 2021). A rapidly changing work environment requires constant innovation and the formation of employees with greater flexibility, efficiency, and faster decision-making (Lamprinou et al., 2021). Traditional leadership is based on strict methodology, oversight, reporting, hierarchy, delegation, and bureaucracy; the agile way is focused on trusting team members and their competencies, working with clients, results, and responding to change (Oruh et al., 2021). Parveen and Adeinat (2019) summarized that the critical task of leaders is to improve the work environment and to be more open to the feedback of their employees. Engaged employees and communication with them are essential for the success of an agile approach. This, in turn, leads to a reduction in employee burnout. According to the studies by Giorgi et al. (2017) and Parveen and Adeinat (2019), leader support reduces work stressors such as role overload, which influences emotional exhaustion. Therefore, the availability of key information reduces burnout’s physical and emotional symptoms (Giorgi et al., 2017). Hence, the following two hypotheses are proposed:

H4: *Availability of key information negatively impacts older employees’ physical burnout symptoms.*

H5: *Availability of key information negatively impacts older employees’ emotional burnout symptoms.*

An effective leader enables age-diverse employees to achieve strategic goals. By acknowledging and responding to the needs of each generation, leaders can maximize the potential of age-diverse employees (Lee et al., 2021). Leaders must consider the differences in attitudes, values, and needs between younger and older employees. By considering the differences between age-diverse employees, leaders with individual measures can increase work engagement and work achievements of older employees (Andel et al., 2012). Intergenerational leadership is becoming an increasingly important factor in the success of companies (Ekoh, 2021). It involves understanding the effects of diversity and introducing behaviors, work practices, and policies that respond to the diversity of the organization (Hsu, 2018). Due to the increasing age diversity in organizations, there is a growing possibility of stereotypes that negatively affect the emotions and behavior of employees, making it difficult for employees to work to their potential and affecting their work efficiency (D’Addio et al., 2010). With the right approach to age diversity, leaders can create an organization in which diverse employees will contribute to success and better results in the workplace (Robson and Hansson, 2007). According to the study by Gill et al. (2010), there is a relationship between leadership style and burnout among older employees. Poor leadership and unclear direction are two main reasons for such burnout (Lee et al., 2021). Accordingly, introducing the appropriate leadership praxes to consider older employees should help prevent different kinds of employee burnout. Therefore, the following two hypotheses are proposed:

H6: *Intergenerational leadership praxes negatively impact older employees’ physical burnout symptoms.*

H7: *Intergenerational leadership praxes negatively impact older employees’ emotional burnout symptoms.*

Leaders who provide essential information about work can better communicate key tasks and intentions for employee actions. Through consistency, they can build better understanding, leading to higher engagement of employees (Avolio and Walumbwa, 2006). Receiving transparent information should reduce the discrepancies between the actual and desired work outcomes. When these are in line, Vogelgesang et al. (2013) reported a positive relationship between leader communication transparency and engagement. This relationship is mediated by follower perceptions of leader behavioral integrity, meaning that concepts’ availability of information, leadership, and engagement are somehow related.

H8: *Availability of key information positively impacts older employees’ work engagement.*

The leader’s stereotypical beliefs or discrimination lead to a lack of opportunities or support for older employees to participate in specific tasks or development activities in the company (Parveen and Adeinat, 2019). Thus, good leadership is a prominent antecedent in companies that facilitate individual and collective efforts to accomplish shared objectives and improve performance through adaptation and innovation (Yukl and Lepsinger, 2006). According to Modesta and Auksë (2016), appropriate leadership reduces employees’ burnout and creates favorable conditions for their professional and personal development. Proper leadership inspires their followers to go beyond self-interest by aligning their values with those of the organization and motivates them to go beyond what is expected of them (Bosak et al., 2021), leading to work engagement among employees (Adisa et al., 2021). Work engagement is defined as a positive and energetic connection with work where engaged employees have a high level of energy, are enthusiastic about their work, and strive to improve the company’s efficiency (Schaufeli et al., 2002). Engaged employees do their job with passion and contribute to the long-term success and improvement of the company. Engaged employees work harder, are more successful, offer better service, and contribute more to profit margins (Schaufeli et al., 2002; Villavicencio-Ayub et al., 2015). They also experience positive emotions such as happiness, joy, and enthusiasm (Demerouti et al., 2015), are in better health, more motivated, and are more creative (Chang et al., 2013). Therefore, the following hypothesis is proposed:

H9: *Intergenerational leadership praxes positively impact older employees’ work engagement.*

### Leadership in Firms of Different Size

New business times require new approaches and ways of leading (Bojadziev et al., 2019). The desire for greater efficiency and competitiveness is the main reason for introducing agile leadership, especially during these challenging times (Attar and Abdul-Kareem, 2020). An agile work environment is characterized by teamwork, a high level of communication, and a low degree of standardization and formalization (Attar and Abdul-Kareem, 2020; McCombs and Williams, 2021). It is almost impossible for companies to grow and expand without effective leadership, as is imperative in an ever-changing market (Bojadziev et al., 2019). While large corporations can survive for a short time without appropriate leadership, the opposite is often true for small businesses (Cong and Thu, 2021). Small companies usually consist of only a few employees and could fail if their leadership structure is compromised (Anning-Dorson, 2021). Small business owners and employers have the power to impact their employees’ mental health in a big way by helping people feel safe, heard, and valued (Cong and Thu, 2021). Accurately providing information about work and changes in a small business leads to the better mental wellbeing of older employees (Bojadziev et al., 2019). Prioritizing mental health keeps employees engaged and increases productivity. Gregersen et al. (2016) found that leaders play a key role in improving the wellbeing of their employees by reducing emotional exhaustion. Communication and leadership become exponentially more important as a company gets larger (Knezović and Drkić, 2021). According to Bojadziev et al. (2019), the democratic style is typical for SMEs. It focuses on group relationships and the sensibility of employees in the company. This leadership style promotes professional competence and the team members take responsibility for their behaviors. The leaders are patient, confident, and friendly (Bojadziev et al., 2019). They guide the members within the group, in which they are part and allow the exchange of ideas from other group members. Such leaders encourage the team members to get involved in the decision-making process (Khan et al., 2015). This results in greater employee satisfaction, motivation, innovation, creativity, and engagement (Bojadziev et al., 2019; Knezović and Drkić, 2021). Also, Bean (2018) found out that employees in smaller companies are less stressed than employees in larger companies. Employees in larger companies reveal that they experience moderate to high levels of work-related stress several times per week. Therefore, the following hypothesis is proposed:

H10: *Firm size moderates the relationships proposed in hypotheses from H1 to H9.*

**FIGURE 1:**
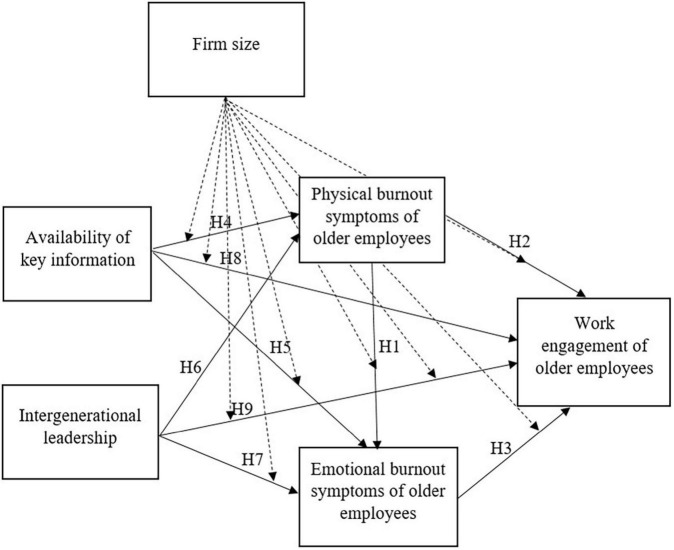
Conceptual model and hypotheses.

According to the above hypothesis, the conceptual model presented in Figure 1 was proposed. Moderating impacts of the firm size are marked with the dotted arrows.

## Materials and Methods

### Sample Type and Data Collection

In this study, a combination of judgmental and quota samples was used. In the first phase, the researchers selected several companies from the representative industries. In the second phase, the quotas were set for employees regarding their age (employees aged from 50 to 55 years, employees aged from 56 to 61 years, and employees aged over 62 years), industry (manufacturing; trade, maintenance, and repair of motor vehicles; financial and insurance activities; professional, scientific and technical activities; information and communication activities; health and social care; real estate business; and catering and other diversified business activities), and company size (small, medium-sized, and large companies). The online questionnaire was addressed to the company’s owner/manager, and the request for data collection was sent *via* e-mail. The owner/manager of the company was asked to distribute a questionnaire among older employees. The response rate of companies prepared to participate in the study was 83%.

### Sample Characteristics

In the survey during the COVID-19 pandemic, 583 older employees participated. The survey included 40.0% of employees aged from 50 to 55 years, 43.0% of employees aged from 56 to 61 years, and 17.0% of employees aged over 62 years. Regarding gender, 53.9% of women and 46.1% of men were involved. The largest share of companies in which older employees are employed was in the large companies (59.2%). Small and medium-sized companies (SMEs) employed 40.8% of employees in our sample. The companies in which older employees are employed were from manufacturing (25.9%); trade, maintenance, and repair of motor vehicles (17.8%); financial and insurance activities (16.6%); professional, scientific, and technical activities (14.1%); information and communication activities (10.2%); health and social care (5.6%); real estate business (4.1%); other diversified business activities (2.3%); catering (2.1%); and other activities (1.3%).

### Measurement Instrument

For measuring the proposed concepts, the Likert-type scale from 1 (strongly disagree) to 5 (strongly agree) was used. Items for the work engagement were adopted from Robinson et al. (2004) and Gallup (2006). Items for the physical and emotional burnout symptoms were adopted from Mosadeghrad (2014). Items for intergenerational leadership were adopted from Naegele and Walker (2006) and Agrawal (2012). Items for the availability of key information were self-generated and partially adapted from Barrett (2006).

### Reliability and Validity of the Scales

Confirmatory factor analysis (CFA) was performed to test the reliability and validity of the scales. Results of the measurement model are presented in Table 1. All standardized indicator loadings ranged from 0.69 to 0.97 and exceeded the suggested threshold of 0.6. Composite reliabilities ranged from 0.94 to 0.96 and are inside the suggested intervals, indicating the adequate reliability of the scales. Average variance extracted (AVE) values varied between 0.71 and 0.93, also showing convergent validity of the scales. Next, the HTMT matrix (Henseler et al., 2015) was used to test the discriminant validity of the scales, and all ratios of correlation between latent variables, except the one between leadership and engagement, are lower than the suggested threshold of 0.85 (Table 2). An additional Fornell and Larcker (1981) test shows that all AVE’s square root calculations were higher than correlations between the constructs.

**TABLE 1:** Indicators means, standard deviations, loadings, and latent variables composite reliabilities and average variances extracted.

Latent and manifest variables	Mean	Std. dev	Lambda	CR	AVE
**Physical burnout**					
My blood pressure varies	2.36	0.994	0.917	0.952	0.869
I’m sweating	2.24	0.996	0.929		
I have vertigo	2.22	0.963	0.950		
**Emotional burnout**					
I am sad	2.24	1.042	0.890	0.944	0.707
I am afraid of losing the job or not finishing the work on schedule	2.54	1.018	0.793		
I feel panic	2.17	0.957	0.891		
I am tense	3.22	1.040	0.695		
I have depressive feelings	2.25	1.047	0.874		
I am emotionally exhausted	2.51	1.133	0.879		
I am quarrelsome	2.24	0.995	0.844		
**Engagement**					
I believe in the successful development and operation of our company	4.07	0.879	0.926	0.958	0.819
I am proud to be employed in this company	3.81	1.037	0.934		
I trust in my colleagues and the manager	4.04	0.914	0.907		
I am aware of the importance of innovation for our company and I am helping to develop the company	4.03	0.923	0.913		
I do my work with passion	3.57	1.033	0.841		
**Intergenerational leadership**					
The leader in our company fosters good relationships between employees	3.60	0.999	0.946	0.970	0.865
The leader in our company fosters good relationships between employees and superiors	3.72	1.000	0.935		
The leader emphasizes and encourages employee motivation in the workplace	3.44	1.029	0.934		
The leader in the company cares that older employees do not feel the negative impact of stereotypes about older employees	3.45	1.029	0.921		
The leader ensures the work satisfaction and wellbeing of employees	3.51	1.078	0.915		
**The availability of key information**					
I have all necessary information to perform my work	4.01	0.889	0.972	0.962	0.926
I have everything I need to carry out my work tasks	4.03	0.866	0.953		

*Fit indices for the measurement model: χ^2^(199) = 731.00; p < 0.001; RMSEA = 0.068; NFI = 0.956; IFI = 0.968; TLI = 0.962; CFI = 0.968.*

**TABLE 2:** HTMT ratio of correlations.

	1	2	3	4
1. Physical burnout				
2. Emotional burnout	0.773			
3. Engagement	0.663	0.804		
4. Intergenerational leadership	0.647	0.786	0.901	
5. The availability of key information	0.624	0.721	0.818	0.841

## Results

Two models were tested in this study. First, a general structural model including the whole sample was proposed, not differentiating between the groups according to the firm size. Then, the participants were divided into two groups, and group analysis was performed.

The structural equation modeling was performed with the maximum likelihood (ML) estimation using the AMOS 27 software. An overall fit assessment resulted in a significant chi-square value [χ^2^(200) = 73,137; *p* < 0.001], which indicates a non-perfect fit. According to Bollen (1989), χ^2^ may be an inappropriate standard when dealing with the complex model and the large sample size, as in our study. Therefore, other fit indices should be used. Accordingly, the following indices were calculated for the general model: root mean square error of approximation (RMSEA) = 0.068, comparative fit index (CFI) = 0.968, tucker lewis index (TLI) = 0.963, and incremental fit index (IFI) = 0.968. All indices were within the accepted boundaries of RMSEA < 0.08, CFI > 0.90, TLI > 0.90, and IFI > 0.90 as proposed by representative authors in the field (MacCallum, 1986; Byrne, 1994; Hu and Bentler, 1999).

The structural model results are presented in Table 3 under “All.” Physical burnout positively influenced emotional burnout (β_1_ = 0.468; *p* < 0.001). Therefore, H1 was supported. Only emotional burnout had a statistically significant negative impact on employee engagement (β_2_ = −0.223; *p* < 0.001). In contrast, the path from physical burnout to engagement was not significant and therefore abandoned in the final model; hence H3 was supported, and H2 was rejected. Both paths leading to physical burnout from the availability of key information (β_3_ = −0.280) and intergenerational leadership (β_4_ = −0.413) were negative and statistically significant at *p* < 0.001, implying that hypotheses H4 and H6 can be supported. In addition, there was a negative and statistically significant impact of the availability of key information (β_5_ = −0.111, *p* < 0.05) and intergenerational leadership (β_6_ = −0.381; *p* < 0.001) on emotional burnout, therefore also H5 and H7 were supported. Both intergenerational leadership (β_7_ = 0.579) and the availability of key information (β_8_ = 0.166) directly positively influence employee engagement at *p* < 0.001. Hence, H8 and H9 were supported.

**TABLE 3:** Structural paths for the entire sample and the two groups (small and large companies).

	All	Sig.	Small	Sig.	Large	Sig.	Hypothesis testing
H1: Physical burnout - > Emotional burnout	**0.468**	*p* < 0.001	**0.633**	***p* < 0.001**	**0.370**	***p* < 0.001**	Supported
H2: Physical burnout - > Engagement	Non-significant in the initial model						Not supported
H3: Emotional burnout - > Engagement	**−0.223**	*p* < 0.001	−0.228	*p* < 0.001	−0.216	*p* < 0.001	Supported
H4: The availability of key information - > Physical burnout	**−0.280**	*p* < 0.001	−0.405	*p* < 0.001	−0.197	*p* < 0.05	Supported
H5: The availability of key information - > Emotional burnout	**−0.111**	*p* < 0.05	**−0.152**	***p* < 0.05**	**−0.032**	**n.s.**	Supported
H6: Intergenerational leadership - > Physical burnout	**−0.413**	*p* < 0.001	−0.329	*p* < 0.001	−0.468	*p* < 0.001	Supported
H7: Intergenerational leadership - > Emotional burnout	**−0.381**	*p* < 0.001	**−0.184**	***p* < 0.01**	**−0.553**	***p* < 0.001**	Supported
H8: The availability of key information - > Engagement	**0.166**	*p* < 0.001	0.181	*p* < 0.01	0.161	*p* < 0.01	Supported
H9: Intergenerational leadership - > Engagement	**0.579**	*p* < 0.001	0.572	*p* < 0.001	0.582	*p* < 0.001	Supported
H10: Moderating impacts	Moderating impacts for relationships proposed in H1, H5, H7						Partially supported

Next in line was the testing for the differences between the groups according to the firm size. Following the recommended practices in the literature (e.g., Steenkamp and Baumgartner, 1998; Vanderberg and Lance, 2000), we tested the measurement invariance across the two groups of large and small companies. This was first used in the measurement and then in the structural model. First, a test of configural invariance or a test of weak factorial invariance was deployed (Horn and McArdle, 1992), where factor loadings were allowed to be free for each of the two groups. All fit indices suggested a good fit of the configural invariance model (Table 4). To test the path differences or path invariance, at least metric equivalence had to be established (Vanderberg and Lance, 2000). Therefore, in the second step, metric invariance or a test of strong factorial invariance was performed to establish whether these factor loadings were invariant across groups.

**TABLE 4:** Invariance test results.

Model	χ ^2^	df	Δχ^2^/df sig.	NFI	IFI	TLI	CFI	RMSEA
**Measurement model**								
*Configural invariance*	1027.96	398		0.940	0.962	0.956	0.962	0.052
Full metric invariance	1047.88	414	0.224	0.938	0.962	0.957	0.962	0.051
*Partial metric invariance*	1043.97	413	0.381	0.939	0.962	0.957	0.962	0.051
**Structural model**								
*Unconstrained paths*	1045.508	415		0.939	0.962	0.958	0.962	0.052
Constrained paths	1073.532	423	0.000	0.937	0.961	0.957	0.961	0.051
*Partially constrained paths*	1050.298	420	0.442	0.938	0.962	0.958	0.962	0.051

The full metric invariance was assessed by constraining all factor loadings across the three groups to be equal. The results in Table 3 indicate that the difference in Δχ^2^/df for configural invariance model and full metric invariance model was not statistically significant at *p* < 0.05, implying that full metric equivalence was achieved. Since at least partial metric invariance must be established for subsequent tests to be meaningful (Steenkamp and Baumgartner, 1998; Vanderberg and Lance, 2000), one factor loading constraint was removed. The path from burnout to “I feel panic” was not invariant across the small and large groups. As can be observed from the invariance table, the partial metric invariance model exhibits an even better fit to the data than the full metric invariance model. The difference in Δχ^2^/df between configural invariance and partial metric invariance models is non-significant.

The same tests were also applied to the structural models. As for the model of unconstrained paths between groups, the model fits the data well. When all paths were constrained to be equal across the groups, the Δχ^2^/df between both models was statistically significant, implying that the constraint path model was not as good as the unconstrained model. In a search for a more valid model with partially constrained paths, the following paths were unconstrained: (a) the path from information to emotional burnout, (b) the path from intergenerational leadership to emotional burnout, and (c) the path from physical burnout to emotional burnout. The final model with partially constrained paths exhibits a better fit than the alternative constrained path model and the same fit as the unconstrained model, with the following fit indices: χ^2^(420) = 1,050.298 (*p* < 0.001), RMSEA = 0.051, CFI = 0.962, TLI = 0.958, and IFI = 0.962.

Bold values in Table 3 indicate the relationships where the differences between both groups exist. According to the results concerning both groups, the impact of the availability of key information on emotional burnout is negative only for small companies, since this path is statistically insignificant for large companies. In contrast, the negative impact of intergenerational leadership on emotional burnout is much stronger in large companies. Concerning the impact of physical burnout on emotional burnout, the positive impact of physical burnout exists in both types of companies but is stronger in small companies. Since only these three relationships were different across the groups, H10 could only be partially supported.

## Discussion

### Theoretical Implications

The COVID-19 pandemic has dramatically increased stress and burnout among older employees (Shipman et al., 2021). Stress and burnout involve both physiological and psychological responses to environmental stressors. This affects the functioning of the autonomic nervous system, which is the primary response system for regulating the body’s physiological reaction to stress (Haar, 2021).

Based on the research, we found that older employees’ physical burnout symptoms positively impact their emotional burnout symptoms, which means that increasing physical burnout symptoms increases emotional burnout symptoms. Moreover, we found that the positive impact of physical burnout symptoms on emotional burnout symptoms of older employees exists in both types of companies but is stronger in small companies, which is in line with Lichtenthaler and Fischbach (2016a,b), Ekoh (2021), and Lee (2021). In addition, Wallo and Kock (2018) emphasized that employees in SME companies may even be more stressed than those who work in other environments. Stress arises in SME company settings from primary sources, namely, overload, uncertainty, understaffing, role conflict, lack of a clear job description, lack of sufficient experience, and personal problems. According to Christian et al. (2011), symptoms of burnout lead to less work engagement among employees. Thus, we found that older employees’ physical burnout symptoms and older employees’ emotional burnout symptoms negatively impact their work engagement. Work engagement is related to the decision and optimal functioning of the wellbeing perceived in the workplace (Christian et al., 2011); therefore, higher levels of stress or burnout of older employees reduce work engagement (Junça Silva and Lopes, 2021).

The availability of key information has a negative impact on older employees’ burnout symptoms, which is evident in large and small companies. Still, especially emotional burnout could be higher in SME companies if key information is not forwarded to older employees. Additionally, agile leadership praxes seems to be more critical for larger companies to prevent burnout due to organizational and occupational hazards of older employees. This could be attributed to the rigidity of large organizations that sometimes forget about the different needs of employees belonging to different generations.

Another implication from our study is that intergenerational leadership praxes negatively impact older employees’ physical burnout symptoms. Intergenerational leadership praxes negatively impact older employees’ emotional burnout symptoms, but this impact is much stronger in large companies. This is in line with Haar’s (2021) claim that employees working in larger-sized firms will report higher levels of burnout risk and that the firm size could be a determinant of burnout rates, reflecting that larger-sized firms operate in more competitive environments, which can create additional pressure on employees.

### Managerial Implications

Therefore, we recommend that companies, especially SMEs, focus on open communication with all employees, which helps to inform better, transfer company goals to employees, improve motivation at work, increase a sense of belonging to the company, encourage employees to express opinions and ideas, and obtain feedback on employees’ wellbeing and management efficiency. In addition, we recommend that companies organize regular meetings (in the case of larger companies, at the level of working groups) where employees are acquainted with work performance, the performance appraisal, the reward system, and plans for the future. At the evaluation meetings, employees can present their opinions, problems, and suggestions for improving the wellbeing and organization of work in the team. We also recommend rotation of employees within the work process, which helps to reduce monotonous and repetitive work that can lead to alienation from work. In this way, employees supplement their knowledge and skills and maintain a higher level of work engagement.

Due to globalization and competitiveness, almost every company changes its infrastructure (Junça Silva and Lopes, 2021). When the authority of any company attempts to change the partial or complete structure, the employees suffer from stress or burnout (Kim et al., 2017). In addition, unclear instructions and expectations, poor listening skills, unreliable data, and lack of collaboration among employees lead to physical and emotional burnout symptoms in older employees and a lower level of work engagement for older employees (Lichtenthaler and Fischbach, 2016a). From this point of view, the availability of information in the company is very important because employees have all the necessary information to perform their work and are constantly informed about changes in the company, which allows them to more easily adapt to changes in the company, especially in SMEs (Bojadziev et al., 2019; Anning-Dorson, 2021). This increases work engagement among older employees (Haley et al., 2013). Additionally, we found that the availability of key information positively impacts older employees’ work engagement. Good information is essential for effective operation and decision-making at all levels of business. Therefore, we recommend that companies of all sizes organize training workshops for leaders where they acquire special knowledge in human resource management such as communication skills, prevention and resolution of interpersonal conflicts, organization of work and distribution of responsibilities, expressing formal and informal praises, and criticisms. In addition, companies should organize work in small groups, which enables the better organization of work, greater transparency in the division of work tasks and responsibilities, and a greater sense of the individual’s information, efficiency, independence, and control over their work. Companies also should use modern communication channels and tools. Using various intelligence systems, managers can communicate quickly and easily with employees. Digitization and reorganization of departments can provide employees with a simplified way of communication, access to documents, information, and superiors.

We discovered that in large companies, employers take care of appropriate leadership because this reduces the emotional symptoms of burnout among older employees (Guérin-Marion et al., 2018; Scheuer and Loughlin, 2021). Ignoring the needs of any age group of employees will likely result in lower productivity and work engagement. According to our study, intergenerational leadership praxes positively impact older employees’ work engagement. As intergenerational workforces dominate today’s labor market, it is more important than ever for leaders to pay special attention to the needs and desires of the different generations in their company to increase employee engagement and business performance (Guérin-Marion et al., 2018). A commitment to understanding the needs of individual employees remains a sound approach to leadership that creates a productive and positive work environment (Hoch, 2014; Guérin-Marion et al., 2018). Diversity is essential for growth in companies, creativity, and innovation because it may be tough to obtain innovative ideas from homogeneous teams who have the same mindset and similar ways of working (Yadav and Lenka, 2020). Demographic change and active aging in the workplace contribute to the creation of new leadership, strategies, and business processes throughout the management of age-diverse employees, especially during the COVID-19 pandemic.

### Limitations and Further Research

This study was only limited to five constructs: physical burnout symptoms, emotional burnout symptoms, work engagement, the availability of key information, and intergenerational leadership. In addition, our research is limited to the time during the COVID-19 pandemic and had a cross-sectional design. The possible pre- and post-COVID-19 analysis would give even more insight into the research constructs and their relationships. Additionally, we would recommend analyzing the differences between older and younger employees. In addition, non-random sampling can present a limitation of our research as well as same-source bias and common method bias. Although some procedural strategies to minimize such biases were deployed, the cause could still be because the respondents evaluated the independent and dependent constructs simultaneously and since the data sources for predictor and criterion variables were the same.

Our research is the first survey in Slovenia that examines physical burnout symptoms, emotional burnout symptoms, work engagement, the availability of key information, and intergenerational leadership among older employees during the COVID-19 pandemic. In addition, our findings highlight the importance of intergenerational leadership and the availability of key information, particularly as teams tend to grow more diverse in current work settings. More and more employees are exposed to various symptoms of burnout, so our research highlights the multiple suggestions about reducing burnout, improving leadership, and increasing work engagement among older employees. The study highlights the importance of agile leadership, diversity in the workplace, work engagement, and information that is increasingly important in today’s business and should be considered. The practical relevance of the study motivates the leaders and academics to promote diversity management practices and increase work engagement in all sizes of companies, especially during the COVID-19 pandemic.

## Data Availability Statement

The raw data supporting the conclusions of this article will be made available by the authors, without undue reservation.

## Author Contributions

BM and MR contributed to conception and design of the study, organized the database, and wrote the first draft of the manuscript and sections of the manuscript. BM performed the statistical analysis. Both authors contributed to manuscript revision, read, and approved the submitted version.

## Conflict of Interest

The authors declare that the research was conducted in the absence of any commercial or financial relationships that could be construed as a potential conflict of interest.

## Publisher’s Note

All claims expressed in this article are solely those of the authors and do not necessarily represent those of their affiliated organizations, or those of the publisher, the editors and the reviewers. Any product that may be evaluated in this article, or claim that may be made by its manufacturer, is not guaranteed or endorsed by the publisher.
